# Recent Developments in the Suzuki-Miyaura Reaction: 2010–2014

**DOI:** 10.3390/molecules20057528

**Published:** 2015-04-24

**Authors:** Irene Maluenda, Oscar Navarro

**Affiliations:** Department of Chemistry, University of Sussex, Brighton BN1 9QJ, UK

**Keywords:** Suzuki-Miyaura, palladium, nickel, N-heterocyclic carbene, phosphine, polymerization

## Abstract

The Suzuki-Miyaura reaction (SMR), involving the coupling of an organoboron reagent and an organic halide or pseudo-halide in the presence of a palladium or nickel catalyst and a base, has arguably become one of most utilized tools for the construction of a C-C bond. This review intends to be general account of all types of catalytic systems, new coupling partners and applications, including the literature between September 2010 and December 2014.

## 1. Introduction

Since its discovery in 1979 [1], the Suzuki-Miyaura reaction (SMR) [2,3,4,5], involving the coupling of an organoboron reagent and an organic halide or pseudo-halide in the presence of a palladium or nickel catalyst and a base, has arguably become one of most utilized tools for the construction of a C-C bond. The reaction follows an oxidative addition transmetallation reductive elimination catalytic cycle that benefits from the use of electron-donating, sterically demanding ligands which promote first and last steps [2]. Clear advantages over other palladium-catalyzed cross-coupling reactions [5] include: (i) mild reaction conditions; (ii) ready availability of organoboron reagents, which also are inert to water and related solvents, as well as oxygen, and generally thermally stable; (iii) tolerant toward various functional groups; and (iv) low toxicity of starting materials and by-products [6]. These features have allowed researchers to utilize it in a wide variety of applications, from natural product synthesis to the development of polymeric materials. Due to its impact in such a variety of fields, Akira Suzuki, together with Richard F. Heck and Ei-Ichi Negishi, was awarded in September 2010 the Nobel Prize in Chemistry “for palladium-catalyzed cross couplings in organic synthesis”. Since then, excellent and thorough reviews covering specific aspects of this reaction have been published: a historical account [7], and reviews focusing on Ni-catalyzed reactions [8], nanocatalysts [9], preformed Pd catalysts [10], sulphur-containing ligands [11,12], and the coupling of polyhalogenated heteroarenes [13]. This review intends to be a more general account of all types of catalytic systems, new coupling partners and applications, including the literature between September 2010 and December 2014. It is worth pointing out that a Scopus search on the topic “Suzuki coupling” lead to more than 3000 articles during that period; therefore, only a selection of the most significant developments and applications of the SMR are presented in here. In addition and for simplicity, details on the specifics of the reaction conditions (base, solvent, temperature) have been omitted unless any of those factors where determinant to the outcome, focusing mostly in coupling partners, ligands, metals and products.

## 2. Catalytic Systems

### 2.1. Homogeneous Systems

#### 2.1.1. Palladium-Based Systems

##### Phosphane-Based Catalytic Systems

The SMR of non-aromatic coupling partners has been featured in several articles. Louie and co-workers devised a protocol for the coupling of heteroaryl boronic acids and vinyl chlorides using Pd(OAc)_2_ and SPhos at 85 °C. Interestingly, any increase or decrease of the temperature led to a lower yield of the desired product and an increase of protodeborilation of the boronic acid [14]. Also using Pd(OAc)_2_, Zhang developed a regioselective and stereospecific coupling of allylic carbonates with arylboronic acids using racemic BINAP as ligand, without added base and under mild reaction conditions [15]. Crudden and co-workers reported on the first regioselective SMR of secondary allylic boronic esters with aryl iodides, using Pd(PPh_3_)_4_ in the presence of Ag_2_O, where the regioselectivity was dictated by the pattern of olefin substitution [16]. More recently and in collaboration with Aggarwal, they reported the first cross-coupling of enantioselective chiral, enantioriched secondary allylic boronic esters, allowing to isolate high yields of major regioisomers by using Pd(dba)_3_, PPh_3_ and Ag_2_O [17].

**Scheme 1:**
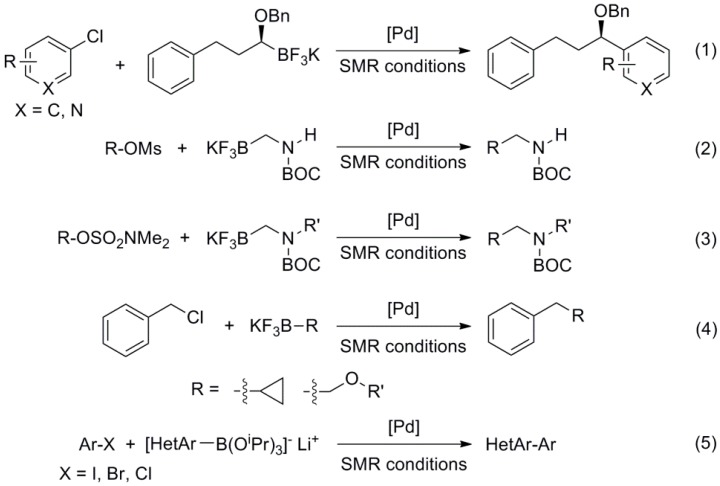
SMR with new organoborane coupling partners.

**Figure 1:**
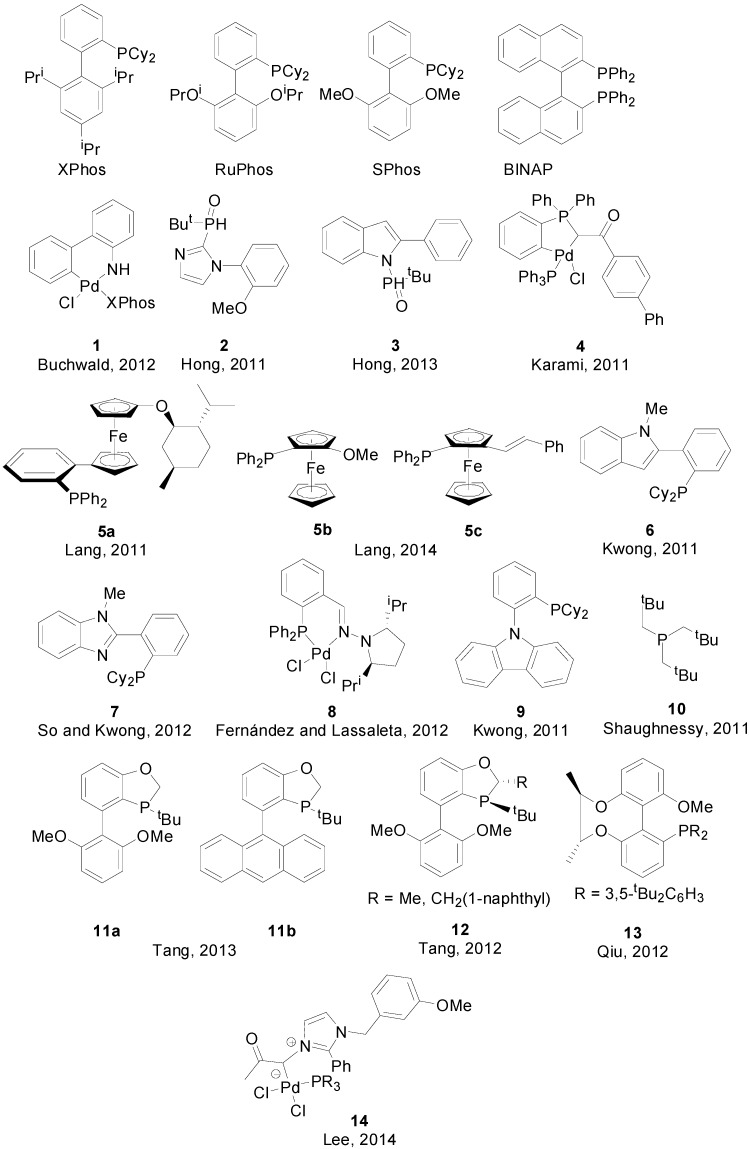
Phosphane-Pd complexes and phosphane ligands used in SMR.

Regarding the use of organoboranes other than the classic boronic acids and esters, Molander reported the coupling of potassium 1-(benzyloxy)alkyltrifluoroborates with aryl and heteroaryl chlorides [18] potassium Boc-protected aminomethyltrifluoroborates with aryl and heteroaryl mesylates [19] and sulfamates [20] and potassium cyclopropyl- and alkoxymethyltrifluoroborates with benzyl chlorides [21], using commercially available phosphines like RuPhos or SPhos (Scheme 1, Reactions (1)–(4)). Buchwald demonstrated that lithium triisopropyl borate salts can be used in SMR, and even devised a one-pot lithiation/borylation/SMR for the coupling of heteroarenes using complex **1** (Figure 1) as pre-catalyst (Scheme 1, Reaction (5)) [22].

Hong reported on the synthesis of two series of secondary phosphine oxide ligands and the use of some of those ligands in combination with Pd(OAc)_2_ (**2** in 2011 and **3** in 2013) for SMR of aryl bromides and chlorides with phenylboronic acid [23,24].

Chelating phosphines and phosphine-bearing palladacycles are also common in SMR. Karami and co-workers prepared ortho-metallated complex **4** for the coupling or aryl bromides and chlorides under mild condition [25]. Lang synthesized chiral *P*,*O*-ferrocene **5a** which, in combination with Pd_2_(dba)_3_ was applied in the coupling of nonactivated aryl bromides with aryl boronic acids at catalyst loadings as low as 1 ppm achieving quite high turnover numbers (TONs) of up to 750,000 [26]. Unfortunately, no atropselective SMR was observed. More recently, in 2014, two new bulky ferrocene supporting ligands **5b** and **5c** allowed for the synthesis of sterically congested tri-*ortho*-substituted byaryls under mild conditions [27,28]. Kwong and coworkers reported on the synthesis of CM-Phos (**6**), used in combination with Pd(OAc)_2_ for the coupling of alkenyl tosylates and mesylates [29]. Later, in collaboration with So, they prepared a 2nd generation of hemilable *P*,*N*-ligands, with **7** affording the best yields for the coupling of aryl mesylates in combination with Pd(OAc)_2_ [30]. Fernández and Lassaletta used complex **8**, bearing a phosphino hydrazone, for asymmetric SMR leading to the formation of enantiomerically enriched biaryls under very mild reaction conditions [31].

**Scheme 2:**
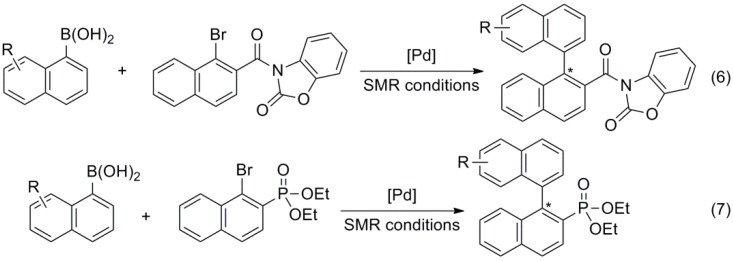
Synthesis of axially chiral biaryls.

Some phosphine-based protocols have been specifically devised for the coupling of sterically challenging substrates. Kwon reported on the synthesis of ligand **9**, used in combination with Pd(OAc)_2_ for the synthesis of tri-*ortho*-substituted biaryls [32]. Di- and tri-*ortho*-substituted biaryls were prepared by Shaughnessy using trineopentylphosphine (**10**) [33]. Ligands **11a** and **11b** were designed by Tang and used for the synthesis of tetra-*ortho*-substituted biaryls by coupling aryl iodides, bromides and chlorides with boronic acids in excellent yields [34]. Previously, this group had reported on the use of similar ligands **12** in the synthesis of axially chiral byaryls in high yields and enantioselectivities, coupling aryl boronic acids with 3-(1-bromo-2-naphthoyl)benzo[d]oxazol-2(3*H*)-one and 1-bromo-2-naphtylphosphonate (Scheme 2) [35]. The same substrate was also the choice of Qiu and co-workers to test ligand **13** [36], and of Suginome and co-workers to test novel helically chiral ligands [37].

Zwitterionic phosphine palladium complex **14**, prepared by Lee and coworkers in 2014, has shown high efficiency in the Suzuki-Miyaura reaction of sterically hindered substrates at room temperature, coupling *ortho*- and di-*ortho*-substituted aryl chlorides, heterocyclic and anthracenyl rings and even performed double arylations in good yields [38].

##### *N*-heterocyclic Carbene (NHC)-Based Systems

**Figure 2:**
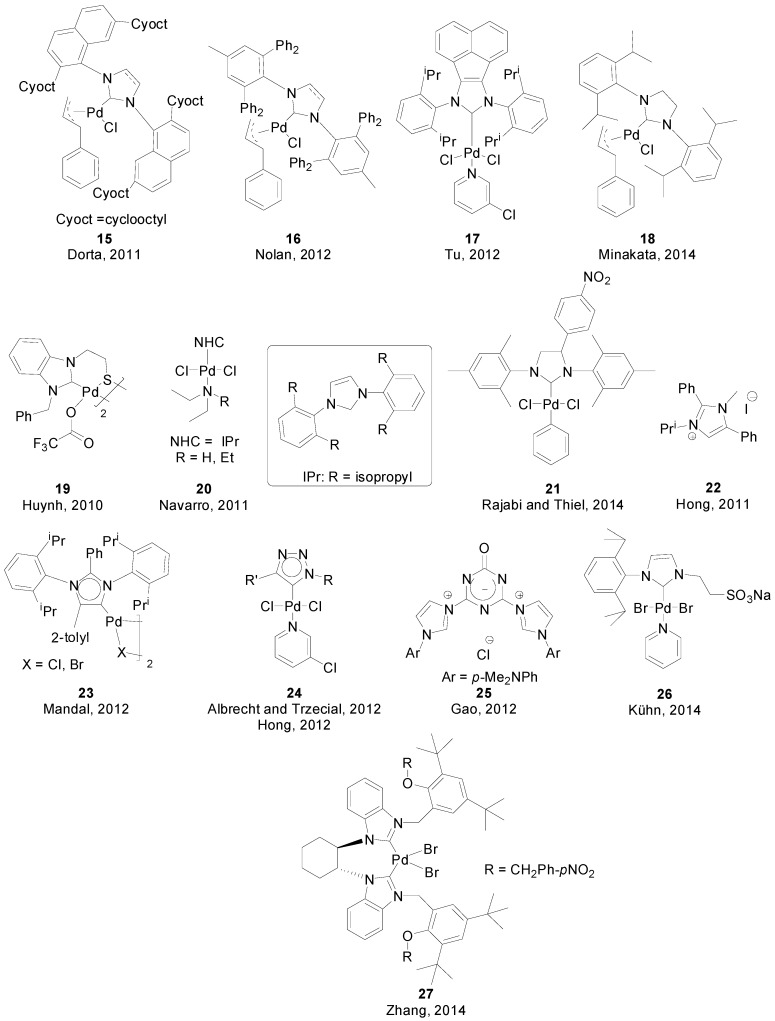
(NHC)-Pd complexes and NHC ligands used in SMR.

Regarding the development of protocols specifically devised for the coupling of sterically challenging substrates, Dorta reported in 2011 the synthesis of complex **15** (Figure 2), which could perform room-temperature Suzuki-Miyaura synthesis of tetra-*ortho*-substituted biaryls.[39] Later, in 2012, Cazin and Nolan reported the synthesis of (IPr^*^)Pd(cinnamyl)Cl] **16**, also featuring a bulky NHC, that performed similar couplings using a lower catalyst loading when compared to Dorta’s [40]. The same year, Tu described the synthesis of complex **17**, which allowed for the synthesis of tetra-*ortho*-substituted biaryls in excellent yields, albeit at higher temperature (80 °C) [41]. Another complex of the same family, [(SIPr)Pd(cinnamyl)Cl] **18**, was applied by Minakata very recently in the stereospecific and regioselective cross-coupling of 2-arylaziridines with arylboronic acids to obtain configurationally defined arylphenethylamine derivatives [42].

Yuan and Huynh prepared a series of dinuclear and tetranuclear Pd(II) complexes of a thiolato-functionalized NHC [43]. Complex **19** was used as catalyst in SMR of aryl bromides using catalyst loadings as low as 0.0025 mol %. Navarro and co-workers reported the synthesis of (NHC)PdCl_2_(TEA) and (NHC)PdCl_2_(DEA) (TEA = triethylamine, DEA = diethylamine) complexes **20** [44,45]. While the TEA-bearing complex exhibited high activity for the coupling of aryl chlorides at room temperature, the DEA-bearing complex required higher reaction temperature (80–90 °C) to match that activity. The authors concluded that while TEA-bearing complex activated faster, leading to the catalytically active species (NHC)-Pd(0), DEA-bearing complexes are more stable due to an rare intramolecular H-bonding between a chlorine and DEA.

Rajabi and Thiel used complex **21** for the cross-coupling of both activated and deactivated aryl chlorides with phenyl, naphtyl and thiopheneboronic acids at room temperature in aqueous medium [46] Hong [47] and Mandal [48] explored the utilization of “abnormal” NHCs (aNHCs) [43] for SMR: [49] while Hong made use of ligand **22** in combination with Pd(OAc)_2_ for the coupling of aryl bromides, Mandal used complexes **23** for the coupling of aryl chlorides at room temperature.

Very similar complexes **24** containing C5-bound 1,2,3-triazolylidene Pd complexes containing a 3-chloropyridine ligand were independently prepared by Albrecht and Trzeciak [50] and Hong [51] in 2012, where the only difference is the identity of the R-substituents: Hong’s had 2,6-di^i^propylphenyl groups while Albrecht explored a wide variety of aryl and alkyl substituents.

Zou and co-workers reported a general protocol for SMR of aryl bromides and chlorides and diarylborinic acids and anhydrides catalyzed by an *in situ*-generated combination Pd/IPr/P(OPh)_3_ in a 1:1:2 ratio [52]. Gao prepared a series of novel bisimidazolium pincer ligands, and **25** was used in combination with Pd(OAc)_2_ for the coupling of aryl bromides using catalyst loadings as low as 0.005 mol% [53].

More recently, Kühn synthesized a sulfonated water-soluble PEPPSI-Pd-NHC **26** complex with good recyclability and performed couplings of aryl chlorides and bromides at room temperature [54]. Zhang prepared a chiral bis-NHC palladium catalyst **27** for asymmetric Suzuki-Miyaura couplings of aryl bromides and chlorides in good yields, observing a strong steric effect of the aromatic substituents on the enantiocontrol of the reaction [55].

##### Other Ligands and Ligandless Systems

**Figure 3:**
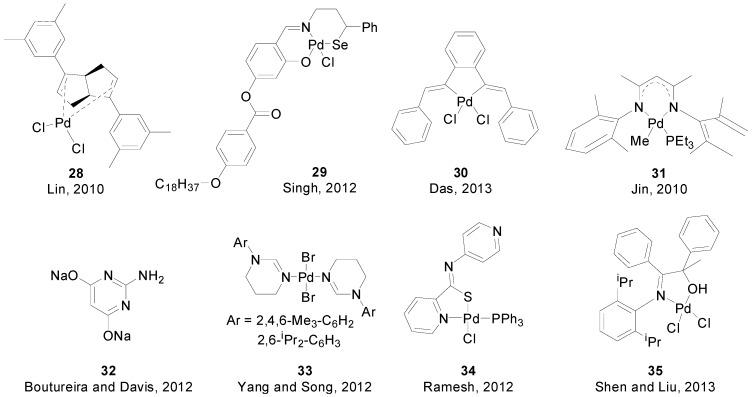
Complexes and ligands used in SMR.

A wide variety of other ligands have been used for SMR. In 2010, Lin and co-workers synthesized chiral complex **28** (Figure 3) and used it, in combination with an extra amount of ligand, for catalytic asymmetric SMR leading to the formation of axially chiral biaryls at room temperature, in excellent yields and good enantioselectivities [56]. Schiff bases have been used as ligands by Singh [57] (**29**) and Das [58] (**30**). While Singh’s was only tested for the coupling of aryl bromides, the latter allowed for the coupling of activated and unactivated aryl chlorides.

**Scheme 3:**
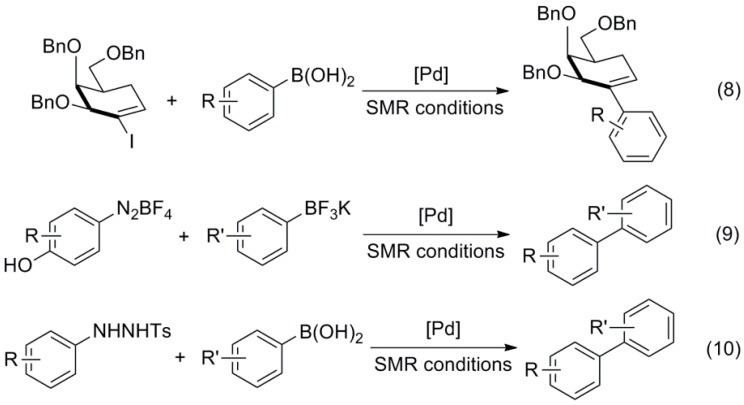
SMR of 2-iodoglycals, phenoldiazonium salts and *N'*-tosylarylhydrazines.

Nitrogen ligands are also very common. In 2011 Jin and Lee synthesized a series of β-diketiminato-phosphine-Pd complexes, being complex **31** the most active for the coupling of mono- and polychlorinated arenes in excellent yields [59]. Boutureira and Davis reported on a general method for the synthesis of 2-arylglycals by coupling 2-iodoglycals with arylboronic acids in aqueous media (Reaction (8), Scheme 3), using L_2_Pd(OAc)_2_ as catalyst (L = **32**) [60]. In 2012, Yang and Song synthesized complexes **33** and used them as catalysts for the coupling of aryl bromides and phenyl chloride with phenyl- and 1-naphthylboronic acid [61]. The same year, Ramesh reported the synthesis of complex **34** for the coupling of deactivated aryl bromides and bromopyridines [62]. More recently, Shin and Liu used complex **35** as catalyst for the coupling of a variety of bromides at mild temperatures (rt—60 °C) [63].

In this period, a series of articles have stressed the advantages and simplicity of ligandless systems, being Pd(OAc)_2_ the catalyst of choice in many cases. For instance, Schmidt used this salt for the coupling of phenoldiazonium salts with potassium trifluoroborates at room temperature [64], while Lu carried out SMR of *N'*-tosylarylhydrazines (Reactions (9), (10), Scheme 3) [65]. In addition, Liu described the synthesis synthesis of heteroaryl-substututed triphenylamine derivatives by coupling heteroaryl halides with 4-(diphenylamino)phenylboronic acid [66].

#### 2.1.2. Nickel-Based Systems

Although Pd-catalyzed protocols have been by far the most investigated, the first example of Ni-catalyzed SMR was reported almost two decades ago by Percec and co-workers [67]. An increasingly growing interest in the use of Ni catalysts has led to important developments in this area [8].

**Scheme 4:**
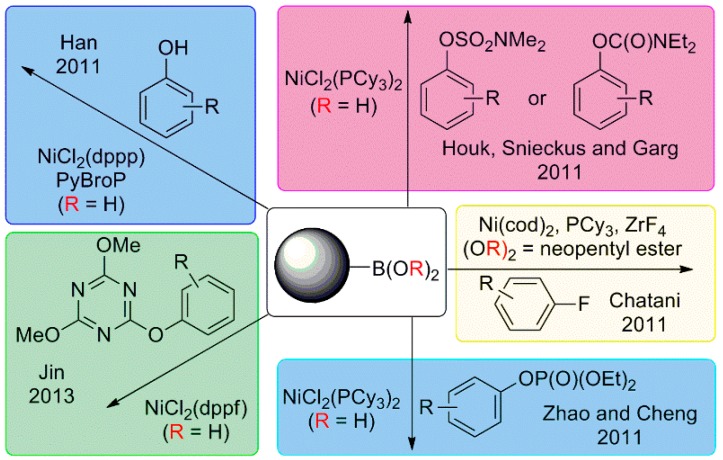
Ni-catalyzed SMR using phosphine ligands.

The most significant feature is the variety of protocols that have been developed to couple other electrophiles than the classic aryl iodides, bromides or chlorides (Scheme 4). Phenols [68,69], carbamates and sulfamates [70,71], fluorides [72], phosphates [73] and heteroaryl ethers [74] have been successfully coupled with organoboron reagents with excellent results. In addition, Hartwig reported on the coupling of heteroaryl boronic acids with heteroaryl halides using low loadings of (dppf)Ni(cinnamyl)Cl under very mild reaction conditions [75].

**Figure 4:**
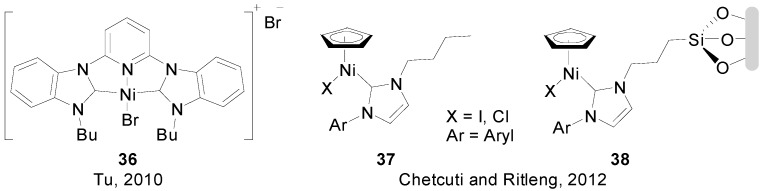
(NHC)_n_Ni(II) complexes used as catalysts in SMR.

A handful of examples of (NHC)-Ni(II) systems have been reported. Although oftentimes their activity in SMR is not particularly extraordinary, they are worth mentioning to account for and promote the research in this subarea. In 2010, Tu and co-workers reported on the use of complex **36** (Figure 4) in combination with PPh_3_ for the coupling or aryl bromides, chlorides, tosylates and mesylates with phenyl boronic acid in good to excellent yields [76]. Radius and co-workers reported on interesting studies on the activation of aryl chlorides and bromides through oxidative addition to (NHC)_2_Ni(0) species [77,78]. More recently, Chetcuti and Ritleng reported on the synthesis of (NHC)Ni(Cp)X complexes **37** and **38** for the coupling of bromo- and chloroarenes [79]. While complex **39** could perform some couplings in low to moderate yields, immobilized complexes **40** displayed a much lower activity.

**Figure 5:**
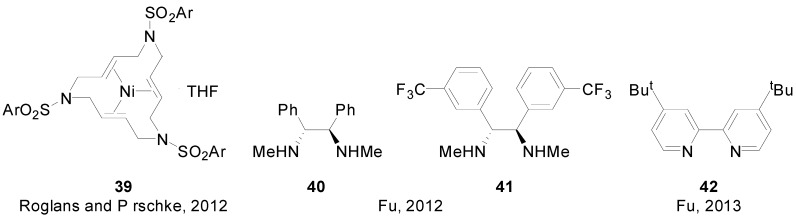
Ni(0) complex and ligands used in Ni-catalyzed SMR.

**Scheme 5:**
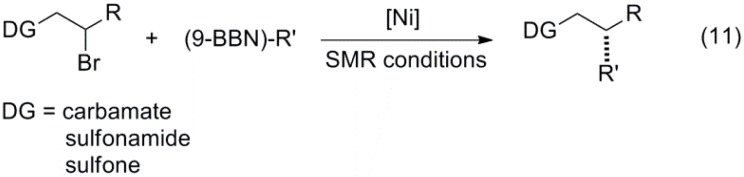
Ni-catalyzed asymmetric coupling of unactivated alkyl electrophiles.

In 2012, Roglans and Pörschke reported on the use of a series of nickel (0) complexes of polyunsaturated azamacrocyclic ligands (**39** and related structures, Figure 5) for the SMR of arylboronic acids and aryl halides [80]. The same year, Fu and co-workers reported the use of carbamates, sulphonamides and sulfones as directing groups in Ni-catalyzed SMR, achieving the asymmetric coupling of unactivated alkyl electrophiles at room temperature (Scheme 5) [81]. The catalyst was formed in situ by combining either ligand **40** or ligand **41** and NiBr_2_·diglyme. Later, the same group reported on the use of ligand **42** in a similar system for the coupling of unactivated tertiary alkyl electrophiles [82].

#### 2.1.3. Iron-Based Systems

Recently, iron has entered the SMR arena. In 2012, Hu and co-workers performed theoretical and experimental studies on the mechanism and regioselectivity of iron-catalyzed direct SMR of pyridine and phenylboronic acid [83]. The authors found that the overall reaction includes three steps, somewhat similar to the regular SMR catalytic cycle: C-H activation, transmetalation, and reductive elimination. The same year, Nakamura and co-workers reported on iron-catalyzed SMR of alkyl bromides with in situ-prepared magnesium tetraalkylborates under very mild reaction conditions [84] and the stereospecific coupling of alkenylboronates and alkyl halides [85].

### 2.2. Heterogeneous Systems

**Figure 6:**
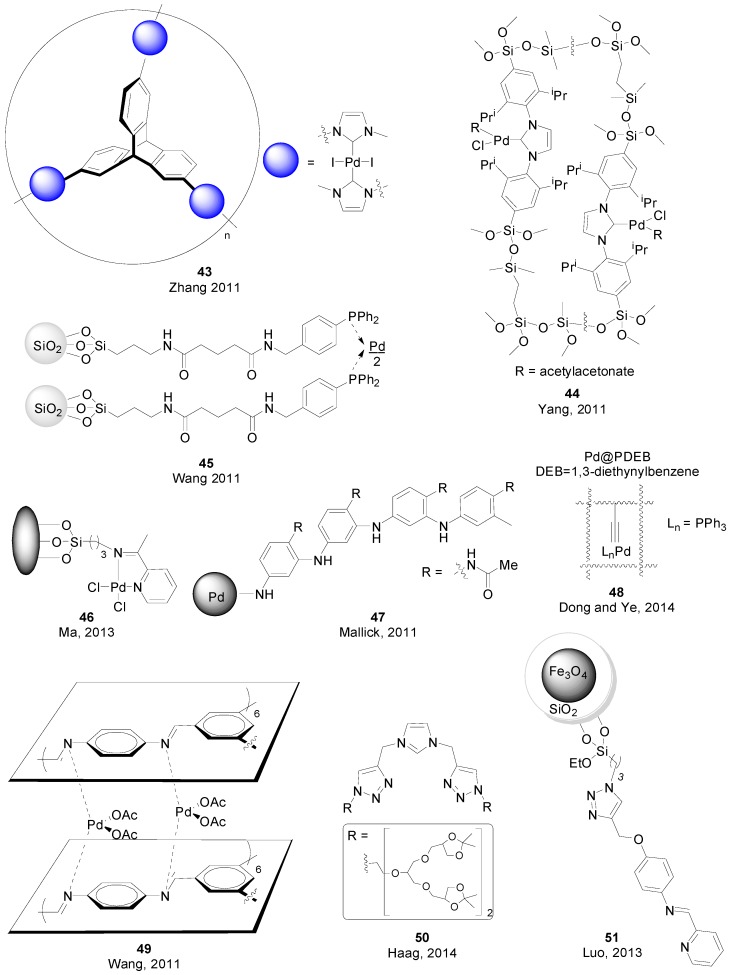
Heterogeneous complexes and ligands for SMR.

Heterogeneous catalysts generally offer the advantage of simple separation and recovery, allowing in many cases for high level of recyclability. With this goal in mind, Yang and co-workers synthesized an NHC-functionalized mesoporous ethane-silica material and a subsequent series of Pd-bearing derivatives, finding that material **43** (Figure 6) could catalyze very challenging couplings involving deactivated and sterically demanding aryl chlorides under relatively mild conditions [86]. Zhang and co-workers prepared NHC-containing, triptycene-based main-chain organometallic polymer **44**, which could be used as a recyclable catalysts for SMR of aryl halides and phenylboronic acid [87]. Silica-supported Pd-phosphine complex **45** was prepared by Wang and used for SMR of aryl iodides, bromides and activated chlorides at room temperature [88]. Very recently, Ma and co-workers synthesized immobilized complex **46**, which was used as catalyst for the coupling of aryl iodides and bromides with phenyl- and 4-methylphenylboronic acid under air atmosphere [89].

Palladium nanoparticles are common in heterogeneous systems and several examples were reported in this period. For instance, Martins and co-workers reported the use of a palladium colloidal solution stabilized with polyvinylpyrrolidone for microwave-assisted SMR of aryl iodides and bromides [90]. Mallick and co-workers synthesized Pd-poly(amino acetanilide) composite **47**, which was used in SMR of aryl and heteroaryl bromides with aryl and heteroaryl boronic acids [91]. Magdesieva and Vorotyntsev reported a series of polypyrrole-Pd nanocomposites that could be recycled when the particles were immobilized in a graphite tissue [92]. Guo and co-workers entrapped Pd nanocrystals in solution-dispersible, poly(*p*-phenyleneethynylene)-based conjugated nanoporous polymer colloids and used them as highly active and recyclable catalysts for the coupling of aryl bromides with phenylboronic acid [93]. Self-encapsulated Pd(II) catalyst **48** was synthesized via cross-linking of 1,3-diethynylbenzene with Pd(OAc)_2_/PPh_3_, by Dong and Ye and used in cross-coupling reactions [94], at ppm to ppb palladium loadings with high recyclability and TONs as high as 3.2 × 10^6^.

In 2011, Wang and co-workers constructed a two-dimensional layered, imine-based covalent organic framework that incorporated Pd(OAc)_2_ between layers **49**. This material was applied to the SMR of aryl iodides and bromides with excellent recyclability [95]. Later in 2014, Haag reported *N*-heterocyclic carbene palladium ligands that were supported on glycerol-dendrons **50**, with the catalytic site at the core position, allowing for cross-coupling reactions to take place with catalyst loadings as low as 0.00001 mol % and high TON values [96].

In recent years, the synthesis of Fe_3_O_4_-supported catalysts that can be separated from the reaction mixture by an external magnet, avoiding laborious separation steps and allowing for an easy reuse, has attracted much attention [97]. Yang and Qin synthesized magnetic silica nanoparticles functionalized with an NHC by co-condensation of IPr carbene-bridged organosilane and tetraalkoxysilane. After coordinating Pd(acac)_2_, these particles were able to catalyze SMR of several aryl bromides and chlorides in isopropanol [98].

Yiang and Sun described the excellent activity of Fe_3_O_4_ and Pd nanoparticles assembled on sulfonated graphene for SMR of aryl bromides and aryl boronic acids [99]. Li and Ma described a method to stabilize Pd(0) on the surface of hollow magnetic mesoporous silica spheres with Fe_3_O_4_ nanoparticles embedded in the mesoporous shell [100], and used this heterogeneous catalyst for the coupling of various aryl halides with phenylboronic acid. Luo and co-workers treated magnetic nanoparticles **51** with a methanolic solution of Na_2_Pd_2_Cl_6_ under reflux conditions to obtain a catalyst that was used for the coupling of aryl bromides and iodides with arylboronic acids in high yields [101]. Very recently, Hyeon and co-workers prepared highly recyclable magnetic hollow nanocomposite catalysts with a permeable carbon surface, which were tested for SMR of aryl iodides and bromides with phenylboronic acid in excellent yields [102].

### 2.3. Reactions in Water

It is estimated that around 80% of the chemical waste from a reaction mixture corresponds to the solvent [103]. From environmental, economic and safety points of view, the use of water as solvent in organic reactions is a clear goal, although challenging and in most cases requiring of high reaction temperatures. In the case of the SMR, the stability of boronic acids in aqueous media gives it an advantage over other cross-coupling reactions to implement water as a solvent.

#### 2.3.1. Homogeneous Systems

**Figure 7:**
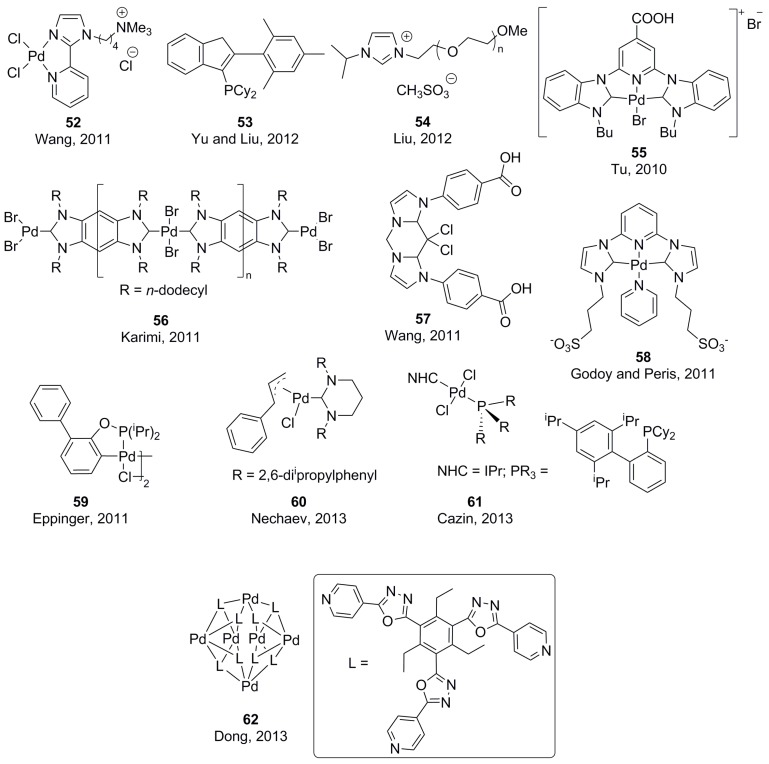
Ligands and complexes for homogeneous SMR in water.

In 2011, Wang and co-workers reported the synthesis of chelating complex **52** (Figure 7) and its application for SMR in air and water at 120 °C, allowing for the coupling of aryl chlorides and bromides in excellent yields [104]. Yu and Liu also reported on the coupling of aryl chlorides using an *in situ* generated catalytic system with Pd(OAc)_2_ and ligand **53**, at 100 °C and in the presence of a quaternary ammonium salt [105]. The same temperature and Pd salt was needed for Liu’s *in situ* system involving the use of NHC precursor **54** for the coupling of mostly bromides [106]. Well-defined NHC-based catalysts for SMR in water were reported by Tu (**55**) [107], Karimi (**56**) [108], Wang (**57**) [109] and Godoy and Peris (**58**) [110]. Karimi reported later on a water-soluble (NHC)-Pd polymer similar to **56**, in which the *N*-groups were replaced by triethylene glycol. This polymer showed very high activity for the coupling of aryl chlorides, together with excellent recyclability [111]. The same year, Eppinger and co-workers used palladacycle **59**, under air and at room temperature, for the coupling of aryl iodides and bromides with a variety of boronic acids [112]. Very recently, Nechaev and co-workers synthesized complex **60**, which was used for the coupling of heteroaryl bromides and chlorides [113]. The same year Cazin and co-workers reported on the synthesis of a series of mixed PR_3_/NHC Pd complexes **61** that were used in SMR of aryl chlorides using very low catalysts loadings and in an aqueous medium (water-isopropanol 9:1) [114]. A self-assembled nano-sized Pd_6_L_8_ (L = 1,3,5-tris(4'-pyrirdyloxadiazol)-2,4,6-triethylbenzene) ball **62** was constructed by Dong for Suzuki-Miyaura coupling reactions at catalysts loadings of ppm [115]. The system allowed to tailor between a homogeneous or heterogeneous format depending on the solubility in different solvents under ambient conditions.

#### 2.3.2. Heterogeneous Systems

In 2010, Huang and co-workers reported on the use of Pd nanoparticles on a porous ionic copolymer of an ionic liquid and divinybenzene for the coupling of aryl bromides and chlorides under air and in water, using Pd loadings as low as 10 ppm [116].

Hu and co-workers embedded Pd nanoparticles in carbon thin film-lined nanoreactors with good results, reproducible even after reusing the catalyst five times [117], while Firouzabadi and Iranpoor used agarose hydrogel to contain the Pd [118]. The use of Pd salts seems to allow for milder reaction conditions: Ma and Lei prepared a heterogenous biopolymer complex wool-PdCl_2_ that performed couplings of aryl chlorides [119] and Li and co-workers used highly recyclable monodispersed zeolitic hollow spheres containing PdCl_2_(pyridine)_2_ to couple aryl bromides and iodides in 60% aqueous ethanol solutions [120]. Bora [121] and Qiu and Liu have recently shown that the solely use of PdCl_2_ and Pd(OAc)_2_, respectively, in the presence of an adequate base can couple a variety of aryl bromides in water [122].

**Figure 8:**
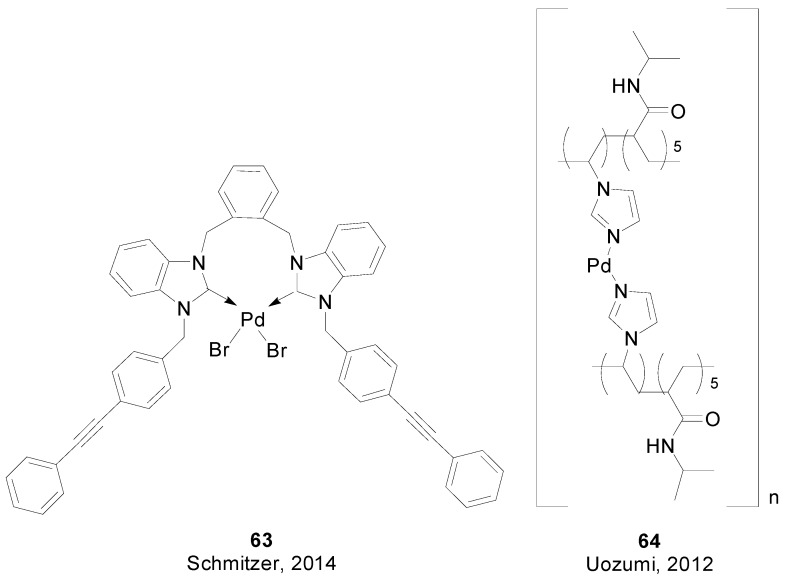
Heterogeneous catalysts for SMR in water.

The use of unsupported Pd-NHC complex **63** (Figure 8) was reported by Schmitzer [123]. This heterogeneous catalyst was used in low loadings for SMR in a green procedure with very good recyclability. Yamada and Uozumi developed a metalloenzyme-inspired polymer catalyst: [124] a self-assembled catalyst of poly(imidazole-acrylamide) and palladium species **64** that promoted the allylic arylation, alkenylation, and vynilation of allylic esters with aryl/alkenylboronic acids in water with catalytic turnover numbers of 20,000–1,250,000.

A few examples of magnetically recoverable systems which undergo reactions in water appeared in 2014. An example was reported by Wand and Li in which a mesoporous magnetic organometallic catalyst promoted SMR in water with high efficiency using as low as 0.002 mol % of Pd (II) [125]. Eight reaction cycles were run without significant decrease in selectivity after which it was recovered by an external magnet.

## 3. Applications

### 3.1. SMR in Natural Products and Drugs Synthesis

**Figure 9:**
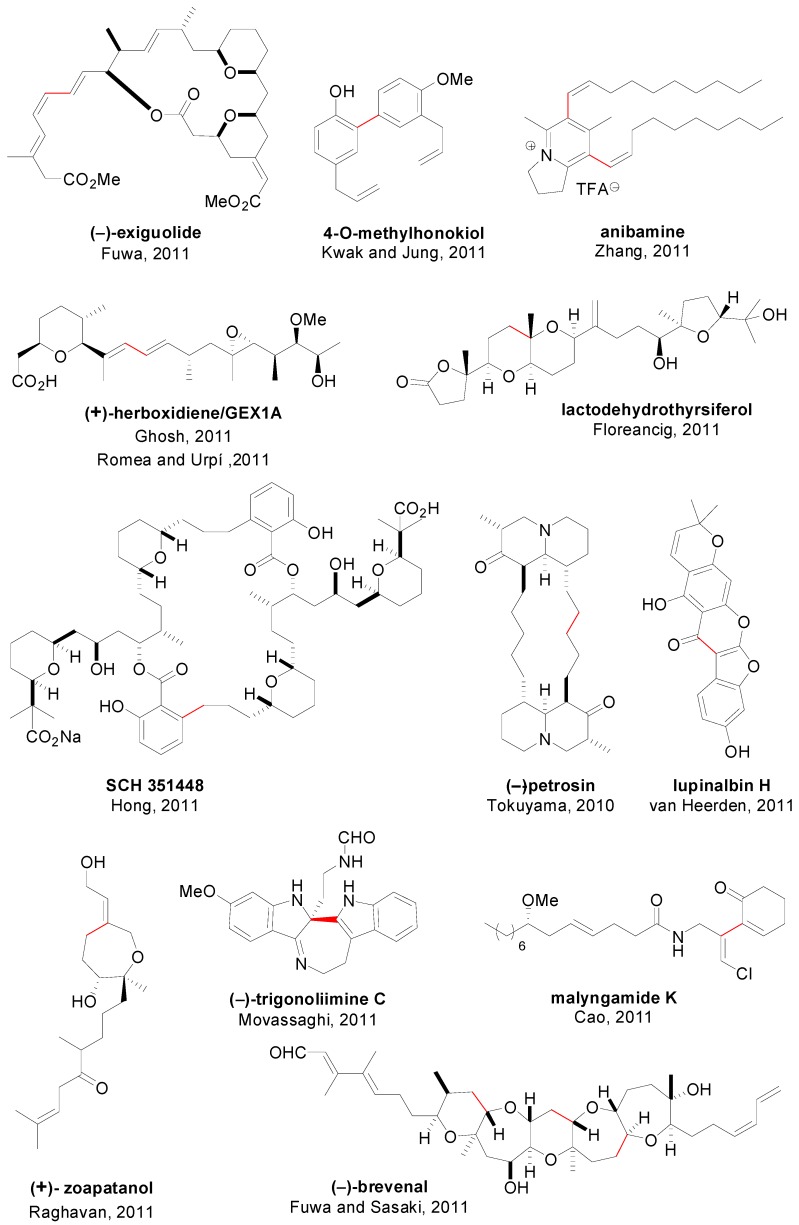
SMR in natural product synthesis (I).

**Figure 10:**
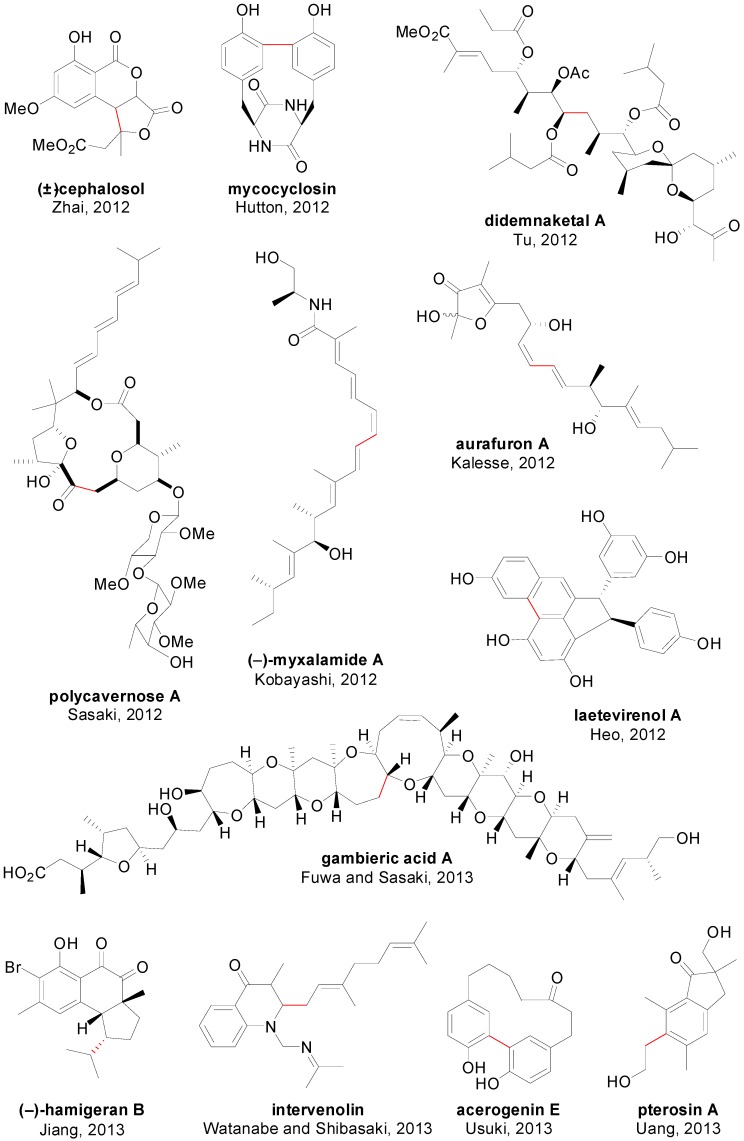
SMR in natural product synthesis (II).

**Figure 11:**
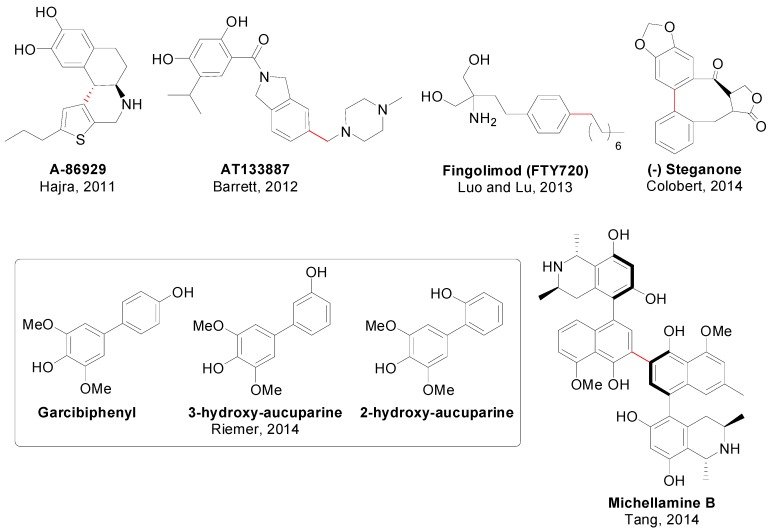
SMR in drug synthesis.

**Figure 12:**
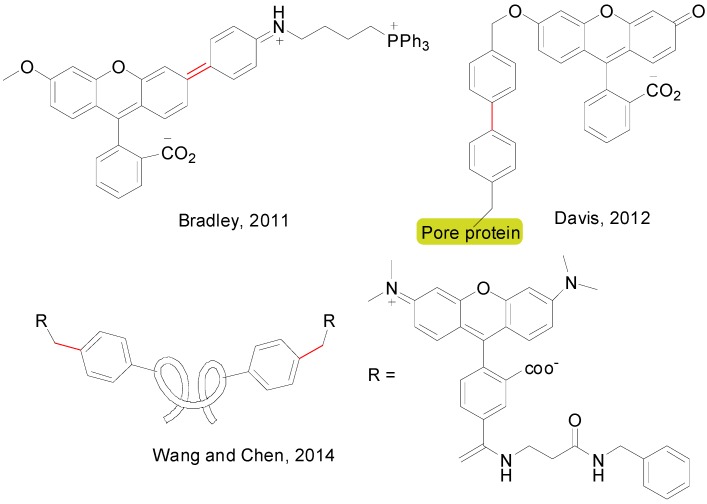
SMR in living organisms.

An account of natural products in which at least one step involved a SMR is depicted in Figure 9 [126,127,128,129,130,131,132,133,134,135,136,137,138] and Figure 10 [139,140,141,142,143,144,145,146,147,148,149,150]. For each compound, the corresponding authors, the year and the bond constructed using a SMR are showed. It is worth of mention that despite the advances in catalyst development in the last 10–15 years, it is rare to find examples in the literature in which the catalyst employed for this often crucial step in the synthesis is different than simply Pd(PPh_3_)_4_, PdCl_2_(dppf) or Pd(OAc)_2_ in combination with PPh_3_ or Ph_3_As [140,144,149,150]. Regarding the use of SMR in drug synthesis only eight examples are shown (Figure 11) [151,152,153,154,155], although this methodology has been used for the synthesis of many libraries of compounds with biological activity [156,157,158]. Some examples have also included SMR for the coupling of amino acids [159] and in chemical transformations in living organisms mediated by synthetic metal complexes (Figure 12), like an intracellular SMR using a cell penetrating Pd[0]-nanocatalyst [160] and the coupling between a modified pore protein with a boronic acid on the surface of *E. coli* [161].

### 3.2. Polymerizations

**Figure 13:**
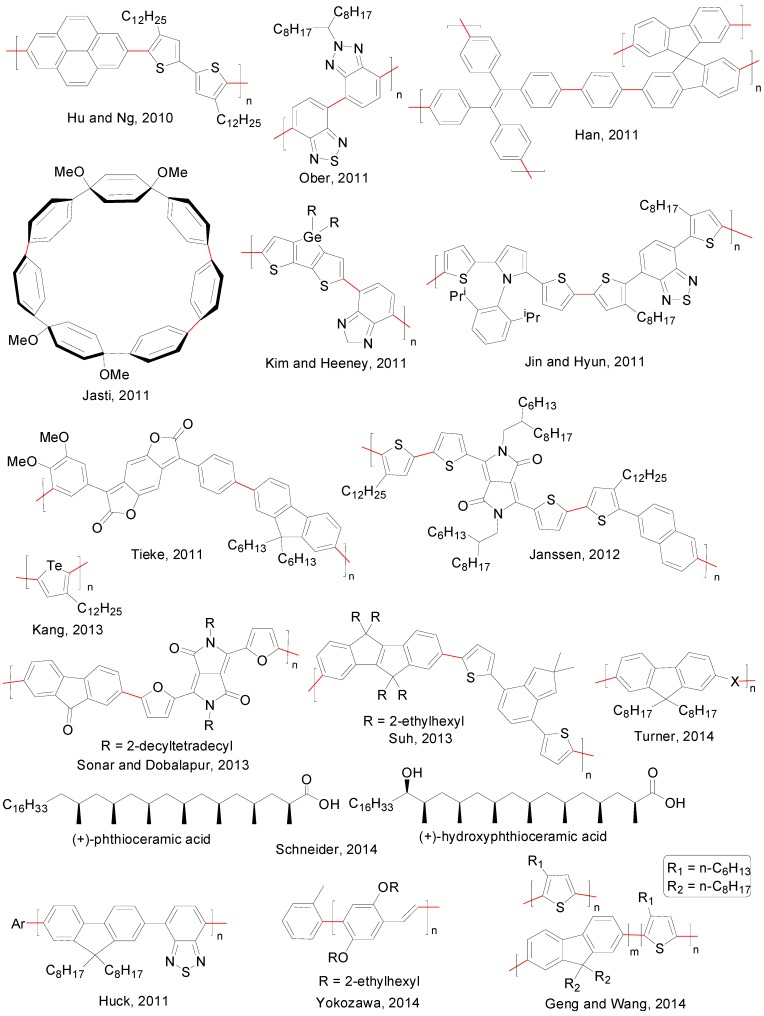
Polymers synthesized using SMP.

The SMR, often referred in this area as “Suzuki polycondensation” (SPC), is a preferred protocol for the synthesis of polyarylenes and related unsaturated polymers [162]. The SPC follows, in most cases [163], a step-growth mechanism with conversions per bond formation step up to 99.99%, making it very attractive for the synthesis of well-defined polymers with specific optical and electronic properties. Both AA/BB and AB polymerizations are common (AA/BB referring to a diboronate coupled with a dihalide, AB referring to a single monomer containing both functionalities), although the first approach is favoured due to an easier functionalization of the monomers. Figure 13 depicts a series of polymers developed since September 2010.

Interestingly, almost all polymers in this list were prepared using Pd(PPh_3_)_4_ or Pd(OAc)_2_ as catalysts [164,165,166,167,168,169,170,171,172], although there are some exceptions [173,174,175,176,177,178]. Focussing more in the catalyst employed and carrying out a catalyst screening for a specific polymerization is gradually becoming more and more common [139,140,141,142,143,144,145,146,147,148,149,150,179,180,181].

## 4. Closing Remarks

Almost thirty five years after its discovery, the Suzuki-Miyaura reaction is more present than ever in the laboratories of researchers from varied disciplines, including both ends of the spectrum: the people that develop new methodology (new catalysts, new coupling partners) and the people that apply it in their field (materials, synthesis, etc.). Although there is a clear disconnection between the newest catalyst development and its application in other areas, fortunately the use of more active and selective catalysts that can couple more challenging substrates, even under milder reaction conditions, is lately becoming more common.
